# Polypharmacy in Older Adults: The Hazard of Hospitalization and Mortality is Mediated by Potentially Inappropriate Prescriptions, Findings From the Moli-sani Study

**DOI:** 10.3389/ijph.2024.1607682

**Published:** 2024-10-24

**Authors:** Simona Costanzo, Augusto Di Castelnuovo, Teresa Panzera, Amalia De Curtis, Stefania Falciglia, Mariarosaria Persichillo, Chiara Cerletti, Maria Benedetta Donati, Giovanni de Gaetano, Licia Iacoviello, the Moli-sani Investigators

**Affiliations:** ^1^ Department of Epidemiology and Prevention, IRCCS NEUROMED, Pozzilli, Italy; ^2^ UOC Governance del Farmaco, Azienda Sanitaria Regionale del Molise -ASREM, Campobasso, Italy; ^3^ Department of Medicine and Surgery, LUM University, Casamassima, Italy

**Keywords:** polypharmacy, potentially inappropriate prescriptions, mortality, hospitalization, elderly

## Abstract

**Objectives:**

We evaluated the impact of polypharmacy on the health of community-dwelling older adults.

**Methods:**

We prospectively analyzed 5,631 individuals from the Moli-sani study (51% men, aged ≥65 years, recruitment 2005–2010, follow-up 2005–2020). Exposure was categorized as chronic polypharmacy therapy (C-PT; ≥5 therapeutic groups and >2 defined daily doses (DDDs)) or non-chronic polypharmacy therapy (NC-PT; polypharmacy but ≤2 DDDs). Hospitalization and mortality were the main outcomes. The mediating role of potentially inappropriate prescriptions (PIP) was examined.

**Results:**

Compared to individuals not on polypharmacy, those in NC-PT and C-PT had higher hazards of mortality [21% (95% CI 7%–37%) and 30% (16%–46%), respectively] and hospitalization [39% (28%–51%) and 61% (49%–75%), respectively]. Similar results were found for cardiovascular outcomes. PIP mediated the association between polypharmacy and outcomes, with mediation effects ranging from 13.6% for mortality to 6.0% for hospitalization. Older adults without multimorbidity experienced the same harm from multiple medications as those with multimorbidity.

**Conclusion:**

Polypharmacy is associated with a higher hazard of mortality and hospitalization, with PIP playing an important role. Addressing “medication without harm” requires assessing the appropriateness of drug prescriptions and monitoring for adverse effects.

## Introduction

Many countries are now super-aged societies where more than 20% of the population is older than 65 years [1]. The demographic changes occurring in the past decades are modifying the prevalence of multiple chronic diseases and the related prescription of multiple medications [1–3], putting a strain on social and healthcare policy planning. Multimorbidity and polypharmacy are the most common conditions managed in geriatric clinical practice. Additionally, older individuals with multimorbidity and related polypharmacy prescriptions, who are at increased risk for adverse events, are the most common users of healthcare and generate high costs [2, 4–6].

Medication consumption among older adults is rising, with rates ranging from 42.5% to 77.0%, estimated to be three times higher than their proportion in the population [6, 7]. The majority of clinical practice guidelines focus on the management of single disease states and do not adequately consider multimorbidity, resulting in long-term treatment with multiple medications [6, 8, 9]. Therefore, prescriptions based on clinical protocols aimed at patient benefit may result in not only favorable but also adverse health outcomes due to unanticipated polypharmacy therapy [10, 11]. Older adults taking multiple medications have been reported to have worse health status compared with those taking fewer medications, and appear to be a vulnerable population [12].

Polypharmacy therapy may sometimes be necessary to manage multiple chronic conditions, but it also poses several risks to the health of older adults and has been found to be associated with various adverse outcomes [13]. In particular, polypharmacy increases the likelihood of inappropriate prescribing and reduces adherence to complex regimens, thereby increasing the risk of morbidity, cognitive and functional impairment, falls and fractures, hospitalizations, and mortality [13–15].

Currently, there is no universally accepted definition of polypharmacy. The most commonly referenced threshold is 5 medications, while higher levels of polypharmacy are often defined by the use of 10 or more medications [16]. Available evidence regarding the efficacy of polypharmacy therapy in the growing elderly population is scarce and rarely derived from real-life conditions, such as those outside hospital settings, but rather from specific clinical contexts [2, 7, 17, 18].

In the framework of the Moli-sani study [19, 20], a large cohort of Italian adults, we prospectively evaluated the impact of polypharmacy therapy on health (hospitalization, length of hospital stay, mortality) in a general population of community-dwelling elderly. In particular, polypharmacy was also considered as a time-varying variable in further survival analyses and we examined whether potentially inappropriate prescriptions (PIP) could potentially mediate the association between polytherapy and poor health.

## Methods

### Study Population

The cohort of the Moli-sani study was randomly recruited from the population of the Molise region through a multistage sampling procedure from the city hall registers [19]. Exclusion criteria were pregnancy at the time of recruitment, institutionalized older adults, impaired understanding or willingness, current poly-trauma or coma, or refusal to sign the informed consent. In total, 30% of subjects could not or refused to participate; these were generally older adults and had a higher prevalence of cardiovascular disease (CVD) and cancer. The Moli-sani study complies with the Declaration of Helsinki and was approved by the ethics committee of the Catholic University of Rome, Italy (P99, A.931/03-138-04, 11 February 2004). All participants provided written informed consent.

The recruitment phase of the Moli-sani cohort was completed in 5 years (2005–2010) and 24,325 subjects [48% men; median (interquartile range, IQR) of age: 54.6 (45.8–64.4) years] were enrolled, of whom 5,831 were older than 64 years [51% men; median (IQR) of age: 71.5 (68.1–76.0) years] were evaluated.

For the present analysis, individuals with incomplete baseline questionnaires (N 183), missing outcome data (N 24) and record linkage to the regional drug prescription register (N 16), were also excluded. The final study sample included 5,631 subjects [51.0% men; median (IQR) of age: 71.4 (68.1–75.8) years].

### Polypharmacy Therapy

During the baseline interview, participants were asked about any prescription medications and to show the boxes of any medications they were using. Name, dose, duration and medication compliance were recorded. A record linkage of the cohort study with the regional drug prescription register allowed for the update of the information on drug therapy for the Moli-sani participants, identifying all prescriptions registered and polypharmacy therapy during the baseline and follow-up periods (until 2020) [21, 22].

Chronic polypharmacy therapy (C-PT) was defined based on the following criteria: a) Number of different therapeutic groups ≥5 and b) Treatments [Number of total defined daily doses (DDDs) of drugs dispensed to the patient in relation to the days of the period > 2DDDs]. Otherwise, no chronic polypharmacy therapy (NC-PT; proxy for non-adherence to polypharmacy), individuals with polypharmacy for point a) but with treatments ≤2 DDDs for point b).

PIP at baseline were evaluated according to the American Geriatrics Society (AGS) Beers Criteria^®^ (AGS Beers Criteria^®^) by using a specific tool implemented in the regional drug prescription register [23, 24].

The AGS Beers Criteria^®^ comprises drugs and drug classes that the AGS and its expert panel consider to be potentially inappropriate medications for use in older adults. The expert panel organized the criteria into five general categories: 1) Medications considered potentially inappropriate; 2) Medications potentially inappropriate in patients with certain diseases or syndromes; 3) Medications to be used with caution; 4) Potentially inappropriate drug–drug interactions; 5) Medications whose dosage should be adjusted based on renal function.

The information available was whether, during the calendar year of enrollment, a participant had taken a drug or group of drugs considered potentially inappropriate, without a detailed breakdown of the specific criteria used to define PIP.

### Outcomes Ascertainment

The main outcomes that occurred in the cohort during follow-up were ascertained by individual-level record linkage to the Molise regional register of deaths (ReNCaM register: “Registro Nominativo delle Cause di Morte”) and hospital discharge records (HDRs). The Moli-sani Study cohort was followed up until 31 December 2020.

#### All-Cause and Cause-Specific Mortality

Cause-specific mortality was assessed using the ReNCaM register, validated by Italian death certificates (ISTAT form), and coded according to the International Classification of Diseases (ICD-9). The primary outcome was all-cause mortality. Additionally, cardiovascular mortality included deaths from diseases of the circulatory system if the underlying cause of death included ICD-9 codes 390–459. Cancer mortality was considered when the underlying cause of death included ICD-9 codes 140–208.

#### All-Cause and Cause-Specific Hospitalizations

The Italian healthcare system follows a single-payer system, and it is based on information reported in the regional register of HDRs, which includes all hospitalizations of all citizens residing in a given region, both in private and public national hospitals. A hospitalization was defined as any length of stay of at least 24 h in a hospital, clinic, emergency room or other similar facility. If a patient was transferred to another hospital or facility, this was considered a single hospitalization. Hospitalizations for the following conditions were excluded: pregnancy complications, childbirth, rehabilitation, and chemotherapy and/or radiotherapy (i.e., elective day care in a hospital-based unit).

Incidence was defined as the first occurrence of a hospitalization for any cause or cause-specific admission. In this context, the primary outcome was all-cause hospitalization.

Hospitalizations for ischemic heart disease (IHD), cerebrovascular events and CVD were defined as reported in Supplementary Table S1. All available hospitalizations for each cohort member during the follow-up period were collected, summing the total number of hospitalizations during the follow-up. Finally, the total number of hospital days for all hospitalizations accrued during follow-up was calculated.

A detailed description of the common risk factors is provided in Supplementary Appendix S1.

### Statistical Analysis

Adjusted survival curves were constructed for all-cause mortality and all-cause hospitalization to show event rates during follow-up by level of polypharmacy therapy.

Hazard ratios (HRs) and 95% CIs for the main outcomes by polypharmacy were calculated using Cox proportional hazards models (unadjusted, age- and sex-adjusted and multivariable) with time-on-study as the time scale and considering No PT as the reference. We defined potential confounders *a priori* and identified them based on existing literature, rather than relying on statistical criteria [25].

To reduce the effect of confounding, the propensity-score method was used. Individual propensities to receive polytherapy (NC-PT or C-PT) were assessed using a multivariable logistic-regression model that included age, sex, education level, income, occupational social class, area of residence total physical activity, smoking, body mass index, history of CVD, general practitioner (GP) diagnosis of hypertension, GP diagnosis of type 2 diabetes, atrial fibrillation, heart failure, history of cancer, pulmonary disease, and chronic kidney disease. Associations between polypharmacy and main outcomes were then appraised by multivariable Cox regression models with the use of propensity scores to account for the inverse probability of polytherapy weighting. The predicted probabilities from the propensity score model were used to calculate the stabilized inverse probability-weighting weight [26]. The stabilized weights were normalized so that they added up to the actual sample size. Two different propensity scores were obtained, one for the NC-PT vs. the No PT comparison and the other for the NC-PT vs. the No PT comparison (Supplementary Figure S1).

The final multivariable Cox proportional hazards model served as the reference for the mediation analysis to estimate the contribution of PIP. For the mediation analysis, we used the publicly available %MEDIATE macro in SAS software, which calculates point and interval estimates of the percentage of the exposure effect (PTE) explained by one or more intermediate variables, with 95% CIs and *P*-values [27].

Additional survival analyses were performed considering the exposure to polypharmacy as a time-varying variable. Subgroup analyses were carried out separately by sex, age classes, education, and history of CVD taking into account the final multivariable Cox proportional hazards model. Multiplicative interaction between the polypharmacy regimen and the designed effect modifier in relation to the main outcomes was tested with cross-product terms.

Furthermore, we tested the association between the polypharmacy regimen and the total number of hospitalizations and hospital days accrued during follow-up using a multivariable Poisson regression model, with adjustment for the observed length of follow-up.

Dummy variables were created for missing values of each categorical variable of interest. A two-sided *P*-value <0.05 was considered statistically significant. Data analysis was performed using SAS/STAT software, version 9.4 (SAS Institute Inc., Cary, NC, United States) [28].

## Results

The study included 5,631 older adults (50.9% men), with a mean baseline age of 72.4 ± 5.4 years. Of those, 1,059 (18.8%) were not taking long-term medications, 1,875 (33.3%) were taking 1–4 medications a day, 1,496 (26.6%) were taking 5–9 medications (polypharmacy), and 1,201 (21.3%) were taking 10 or more medications (heightened polypharmacy).

In total, 29% of the older adults were on chronic polytherapy [C-PT; median number of drugs 10 (IQR: 8–13)], 18.9% were on non-chronic polytherapy [NC-PT; 7 (6–9)], while 52.1% were classified as not on polytherapy [No PT; 1 (0–2)].

Supplementary Tables S2, S3 show the distribution at baseline of the main characteristics, the most common chronic degenerative diseases in older adults according to not being on polypharmacy or being on polypharmacy therapy. Individuals on polypharmacy (both NC-PT and C-PT) were older than those not on polypharmacy (*P*-value < 0.0001), had lower levels of education and social status in childhood, and were more frequently retired (*P*-value = 0.0001, 0.0019 and 0.0013, respectively; Supplementary Table S2). Moreover, they had lower levels of physical activity, ate fewer calories, and drank less alcohol, being more frequently abstainers or ex-drinkers. However, they did not differ in their adherence to the Mediterranean diet. Women were more represented in the NC-PT group in comparison to the other groups (*P*-value <0.0001). There were no significant sociodemographic differences in terms of marital status, place of residence and income between individuals not on polypharmacy therapy and those on C-PT. In general, individuals on polypharmacy therapy showed a higher prevalence of multimorbidity, chronic-degenerative diseases and obesity, but a lower prevalence of liver disease, Parkinson’s disease and Alzheimer’s disease (Supplementary Table S3).

During a median follow-up of 12.6 years (IQR 10.6–13.8 years; 64,716 person-years), a total of 2,001 deaths (41.1% for CVD and 28.7% for cancer) were ascertained; additionally, 4,342 hospital admissions (56.6% for CVD, 14.2% for IHD and 16.2% for cerebrovascular disease) occurred.

**FIGURE 1:**
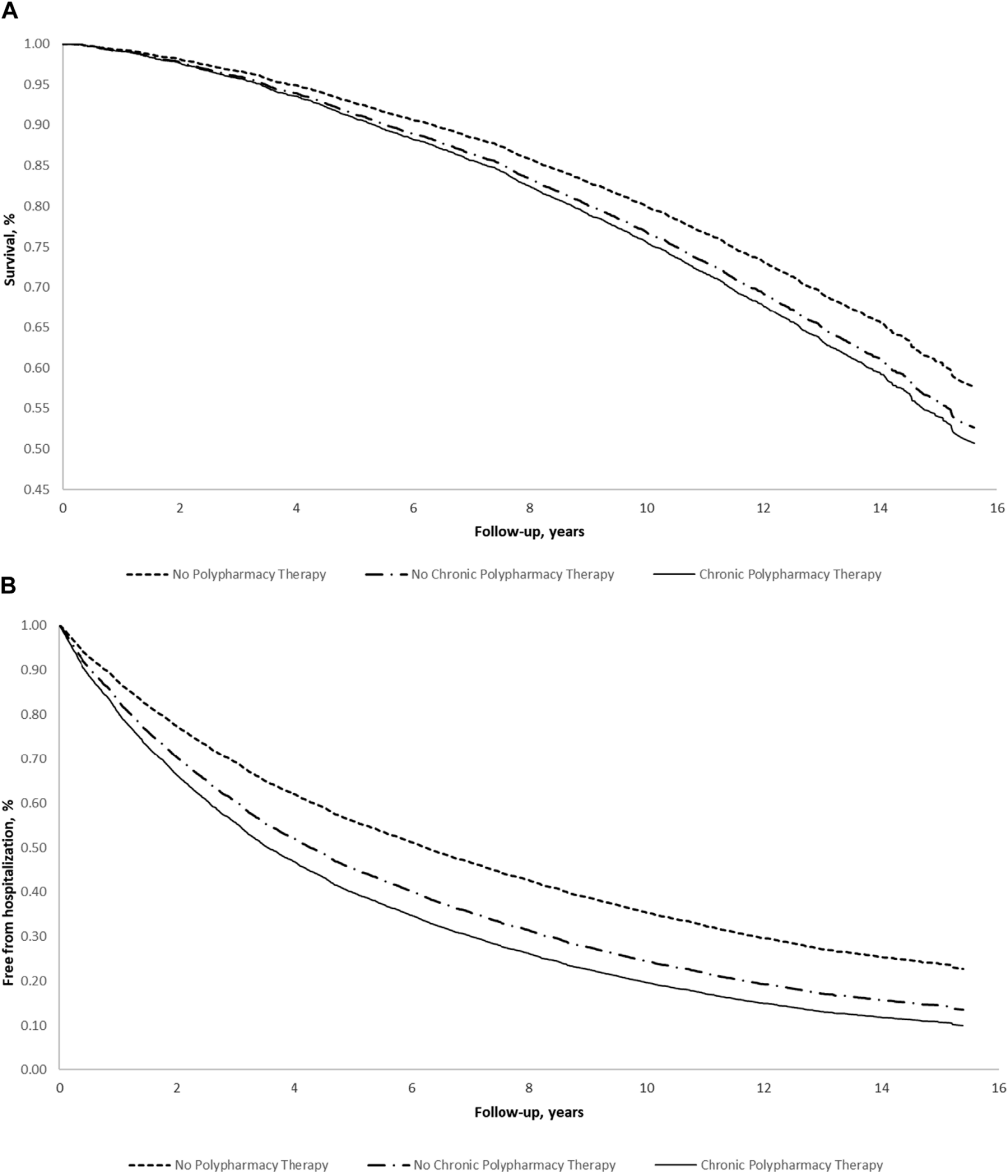
Multivariable survival estimates for **(A)** all-cause mortality and **(B)** all-cause hospitalization according to polypharmacy in the elderly of the Moli-sani study (N = 5,631) (Italy, 2005–2010). Multivariable survival curves were obtained from the multivariable model adjusted for age, sex, education level, income, occupational social class, area of residence, total physical activity, smoking, body mass index, history of cardiovascular disease, general practitioner diagnosis of hypertension, general practitioner diagnosis of type 2 diabetes, atrial fibrillation, heart failure, history of cancer, pulmonary disease, and chronic kidney disease.

Figure 1 shows the adjusted survival curves (a: for all-cause mortality; b: for all-cause hospitalization) according to polypharmacy. Both figures show that individuals on polypharmacy (both NC-PT and C-PT) had a lower probability of survival or being free from hospitalization for any cause, during follow-up.

Tables 1, 2 report the hazard for all-cause, CVD and cancer mortality according to polypharmacy. Additionally, the same analyses were reported for all-cause and CVD, IHD and cerebrovascular hospitalizations.

**TABLE 1:** Hazard ratio (95% confidence interval) for all-cause mortality and all-cause hospitalization according to polypharmacy in the elderly of the Moli-sani study (N = 5,631) (Italy, 2005–2010).

	No polypharmacy therapy	No chronic polypharmacy therapy	Chronic polypharmacy therapy	*P*-value	P-value for trend
N	2,934	1,063	1,634		
	All-cause mortality				
Person Years	35,236	11,896	17,584		
Number of events (rate %)	819 (27.9)	408 (38.4)	774 (47.4)		
Model not adjusted	Ref.	1.51 (1.34–1.70)	1.99 (1.80–2.19)	<0.0001	<0.0001
Multivariable Model 1	Ref.	1.30 (1.16–1.47)	1.62 (1.46–1.79)	<0.0001	<0.0001
Multivariable Model 2	Ref.	1.21 (1.07–1.37)	1.30 (1.16–1.46)	<0.0001	<0.0001
	All-cause hospitalization				
Person Years	21,307	5,939	7,217		
Number of events (rate %)	2,059 (70.2)	861 (81.0)	1,422 (87.03)		
Model not adjusted	Ref.	1.47 (1.36–1.59)	1.95 (1.82–2.09)	<0.0001	<0.0001
Multivariable Model 1	Ref.	1.45 (1.33–1.57)	1.86 (1.73–1.99)	<0.0001	<0.0001
Multivariable Model 2	Ref.	1.39 (1.28–1.51)	1.61 (1.49–1.75)	<0.0001	<0.0001

Model 1: adjusted for age and sex. Model 2: as Model 1 further adjusted for education level, income, occupational social class, area of residence, total physical activity, smoking, body mass index, history of cardiovascular disease, general practitioner diagnosis of hypertension, general practitioner diagnosis of type 2 diabetes, atrial fibrillation, heart failure, history of cancer, pulmonary disease and chronic kidney disease.

**TABLE 2:** Hazard ratio (95% confidence interval) for cardiovascular and cancer mortality according to polypharmacy in the elderly of the Moli-sani Study (N = 5,631) (Italy, 2005–2010).

	No polypharmacy therapy	No chronic polypharmacy therapy	Chronic polypharmacy therapy	P-value	P-value for trend
N	2,934	1,063	1,634		
	Cardiovascular mortality				
Person Years	35,236	11,896	17,584		
Number of events (rate %)	274 (9.4)	161 (15.2)	375 (23.1)		
Model not adjusted	Ref.	1.79 (1.47–2.17)	2.89 (2.48–3.38)	<0.0001	<0.0001
Multivariable Model 1	Ref.	1.43 (1.18–1.74)	2.23 (1.91–2.61)	<0.0001	<0.0001
Multivariable Model 2	Ref.	1.25 (1.03–1.53)	1.52 (1.27–1.82)	<0.0001	<0.0001
	Cancer mortality				
Person Years	35,236	11,896	17,584		
Number of events (rate %)	279 (9.6)	121 (11.5)	165 (10.2)		
Model not adjusted	Ref.	1.31 (1.06–1.62)	1.22 (1.01–1.48)	0.021	0.022
Multivariable Model 1	Ref.	1.27 (1.02–1.58)	1.10 (0.90–1.33)	0.091	0.25
Multivariable Model 2	Ref.	1.25 (1.00–1.56)	1.03 (0.82–1.28)	0.12	0.64
	Cardiovascular hospitalization				
Person Years	29,314	9,164	11,374		
Number of events (rate %)	1,018 (34.7)	473 (44.5)	972 (59.5)		
Model not adjusted	Ref.	1.48 (1.33–1.65)	2.43 (2.22–2.65)	<0.0001	<0.0001
Multivariable Model 1	Ref.	1.43 (1.28–1.60)	2.27 (2.08–2.49)	<0.0001	<0.0001
Multivariable Model 2	Ref.	1.29 (1.15–1.45)	1.70 (1.53–1.88)	<0.0001	<0.0001
	Ischemic heart disease hospitalization				
Person Years	34,016	11,277	15,892		
Number of events (rate %)	230 (7.8)	110 (10.4)	275 (16.8)		
Model not adjusted	Ref.	1.44 (1.15–1.81)	2.55 (2.14–3.04)	<0.0001	<0.0001
Multivariable Model 1	Ref.	1.63 (1.30–2.05)	2.64 (2.21–3.16)	<0.0001	<0.0001
Multivariable Model 2	Ref.	1.44 (1.14–1.82)	1.66 (1.35–2.05)	<0.0001	<0.0001
	Cerebrovascular hospitalization				
Person Years	33,902	11,319	16,452		
N of events (rate %)	299 (10.2)	137 (12.9)	269 (16.5)		
Model not adjusted	Ref.	1.38 (1.13–1.69)	1.87 (1.59–2.21)	<0.0001	<0.0001
Multivariable Model 1	Ref.	1.27 (1.03–1.56)	1.66 (1.41–1.97)	<0.0001	<0.0001
Multivariable Model 2	Ref.	1.20 (0.98–1.48)	1.39 (1.15–1.69)	0.0033	0.0007

Model 1: adjusted for age and sex. Model 2: as Model 1 further adjusted for education level, income, occupational social class, area of residence, total physical activity, smoking, body mass index, history of cardiovascular disease, general practitioner diagnosis of hypertension, general practitioner diagnosis of type 2 diabetes, atrial fibrillation, heart failure, history of cancer, pulmonary disease, and chronic kidney disease.

Considering the individuals not on polypharmacy as a reference and after adjusting for possible confounders (model 2, Table 1), the individuals in NC-PT and C-PT showed a higher hazard of mortality [21% (95% CI 7%–37%) and 30% (16%–46%), respectively, *P*-value < 0.0001) and all-cause hospitalization [39% (28%–51%) and 61% (49%–75%), respectively, *P*-value < 0.0001)]. Similar trends were found for all secondary outcomes (CVD and cancer mortality, and CVD, IHD, and cerebrovascular hospitalizations), except for cancer mortality in C-PT elderly and cerebrovascular hospitalization in NC-PT elderly (Table 2).

Additionally, a propensity score approach was used to control for residual confounding by comorbidities. The unadjusted and propensity score-adjusted differences in NC-PT vs. No PT and C-PT vs. No PT for each variable included in the propensity score are shown in Supplementary Figure S1; the c-statistic of the propensity score models was 0.68 and 0.83, respectively. All the pre-treatment differences disappeared after adjustment by propensity score weighting. Using the propensity score method (Table 3), the association between the studied health outcomes and polypharmacy did not differ from the previous results, mainly for the primary outcomes and for C-PT elderly overall (Tables 1, 2).

**TABLE 3:** Hazard ratio (95% confidence interval) for the studied outcomes with polypharmacy in the elderly of the Moli-sani Study (N = 5,631), considering the propensity score method (Italy, 2005–2010).

	No polypharmacy therapy	No chronic polypharmacy therapy	*P*-value
All-cause mortality	Ref.	1.15 (1.01–1.30)	0.029
Cardiovascular mortality	Ref.	1.17 (0.95–1.44)	0.15
Cancer mortality	Ref.	1.16 (0.93–1.44)	0.19
All-cause hospitalization	Ref.	1.39 (1.28–1.50)	<0.0001
Cardiovascular hospitalization	Ref.	1.30 (1.17–1.46)	<0.0001
Ischemic heart disease hospitalization	Ref.	1.44 (1.15–1.80)	0.0017
Cerebrovascular hospitalization	Ref.	1.19 (0.97–1.47)	0.092

	No polypharmacy therapy	Chronic polypharmacy therapy	*P*-value
All-cause mortality	Ref.	1.42 (1.29–1.57)	<0.0001
Cardiovascular mortality	Ref.	1.62 (1.38–1.89)	<0.0001
Cancer mortality	Ref.	1.17 (0.97–1.40)	0.093
All-cause hospitalization	Ref.	1.70 (1.59–1.82)	<0.0001
Cardiovascular hospitalization	Ref.	1.74 (1.59–1.90)	<0.0001
Ischemic heart disease hospitalization	Ref.	1.83 (1.53–2.20)	<0.0001
Cerebrovascular hospitalization	Ref.	1.50 (1.27–1.77)	<0.0001

Controlling for age, sex, education level, income, occupational social class, area of residence, total physical activity, smoking, body mass index, history of cardiovascular disease, general practitioner diagnosis of hypertension, general practitioner diagnosis of type 2 diabetes, atrial fibrillation, heart failure, history of cancer, pulmonary disease and chronic kidney disease.

Supplementary Table S4 shows the distribution of the total number of hospitalizations and the total number of hospital days accrued during follow-up for all-cause, CVD, IHD and cerebrovascular hospitalizations. The 15,161 total multiple hospital admissions had a median duration of 20 days (IQR: 9–41). Compared to individuals not on polytherapy, those on NC-PT and C-PT accumulated significantly more total hospitalizations (22%, 95% CI: 17%–28% and 50%, 44%–57%, respectively; Supplementary Table S5) and total hospital days (14%, 12%–15% and 15%, 13%–16%, respectively). Similar trends were observed when considering the total number of hospitalizations for CVD and IHD and the total number of hospital days for cardiovascular hospitalizations. The observed inverse trend relative to the total number of hospital days for cerebrovascular hospitalizations should be further investigated (Supplementary Table S5).

**FIGURE 2:**
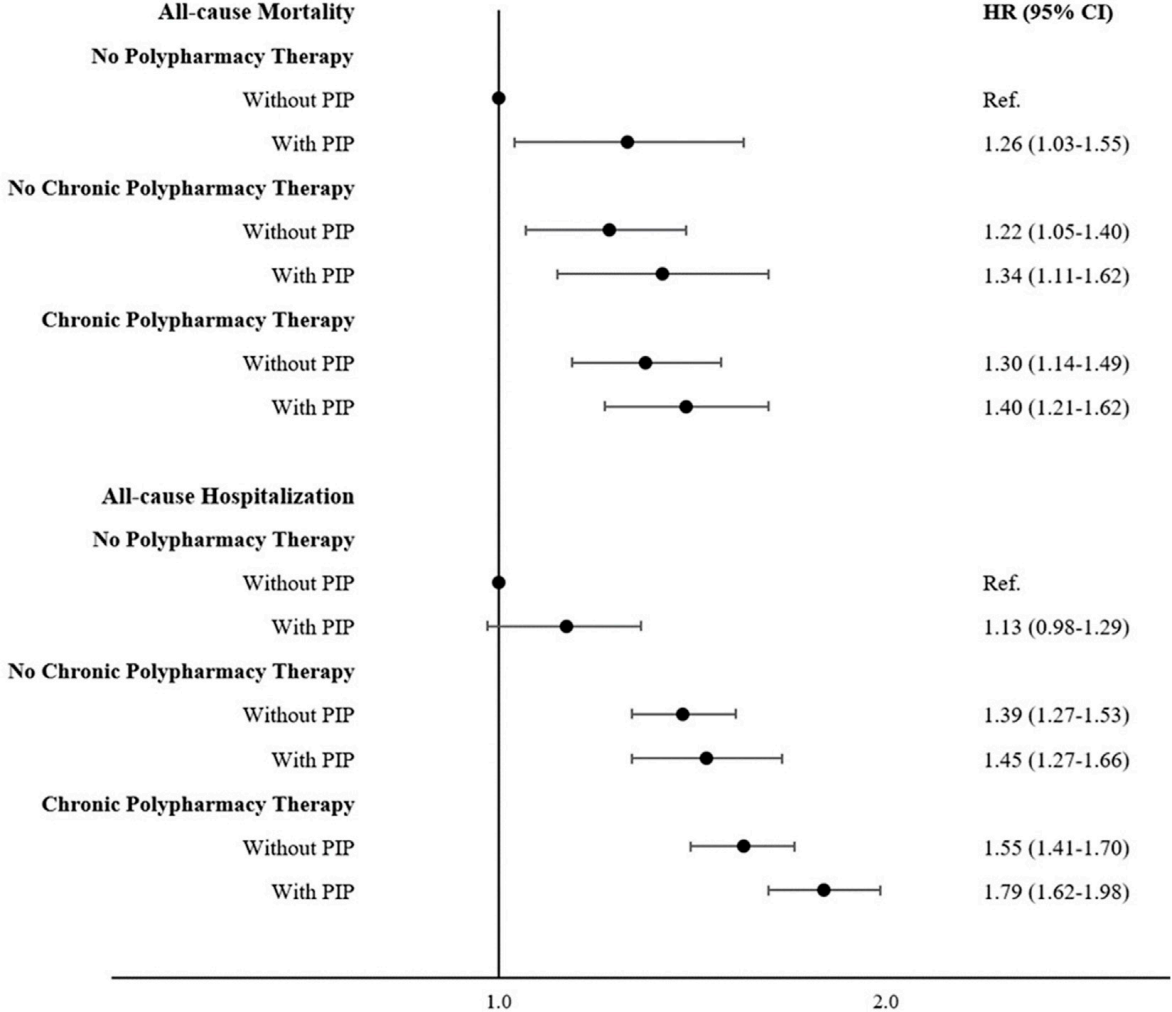
Role of the potentially inappropriate prescriptions in the relationship between polypharmacy and main outcomes (Moli-sani Study, Italy, 2005–2010). Multivariable survival curves were obtained from the multivariable model adjusted for age, sex, education level, income, occupational social class, area of residence, total physical activity, smoking, body mass index, history of cardiovascular disease, general practitioner diagnosis of hypertension, general practitioner diagnosis of type 2 diabetes, atrial fibrillation, heart failure, history of cancer, pulmonary disease, and chronic kidney disease.

The prevalence of PIP was 11.1%, 29.1% and 43.3% in the No PT, NC-PT and C-PT groups, respectively. Figure 2 and Supplementary Table S6 show that individuals with PIP had a higher risk of all-cause mortality and hospitalizations. Additionally, Table 4 shows that potentially inappropriate prescriptions are important mediators of the association between polypharmacy therapy and all-cause mortality, with PTE being 11.6% and 13.6% for NC-PT and C-PT, respectively. Similar results were found for cardiovascular mortality and hospitalizations (Table 4). The PIP mediation effects for all-cause hospitalization were slightly inferior (5.9% and 6.0% for NC-PT and C-PT, respectively).

**TABLE 4:** Mediation analysis by potentially inappropriate prescriptions. Hazard ratio (95% confidence interval) for the studied outcomes with polypharmacy therapy without and with the mediator (Moli-sani Study, Italy, 2005–2010).

	No polypharmacy therapy	No chronic polypharmacy therapy	Chronic polypharmacy therapy
N	2,934	1,063	1,634
% PIP	11.1	29.1	43.3
All-cause mortality			
Not adjusted for the mediator	Ref.	1.21 (1.07–1.37)	1.30 (1.16–1.46)
Adjusted for the mediator: PIP	Ref.	1.17 (1.04–1.32)	1.26 (1.12–1.43)
PTE		11.6%; *P* = 0.015	13.6%; *P* < 0.0001
All-cause hospitalization			
Not adjusted for the mediator	Ref.	1.39 (1.28–1.51)	1.61 (1.49–1.75)
Adjusted for the mediator: PIP	Ref.	1.35 (1.24–1.46)	1.56 (1.44–1.69)
PTE		5.9%; *P* = 0.0026	6.0%; *P* < 0.0001
Cardiovascular mortality			
Not adjusted for the mediator	Ref.	1.25 (1.03–1.53)	1.52 (1.27–1.82)
Adjusted for the mediator: PIP	Ref.	1.22 (1.00–1.50)	1.48 (1.21–1.81)
PTE		13.2%; *P* = 0.020	12.4%; *P* = 0.0001
Cardiovascular hospitalization			
Not adjusted for the mediator	Ref.	1.29 (1.15–1.45)	1.70 (1.53–1.88)
Adjusted for the mediator: PIP	Ref.	1.24 (1.10–1.39)	1.61 (1.44–1.80)
PTE		12.3%; *P* = 0.0002	10.0%; *P* < 0.0001

Model adjusted for age, sex, education level, income, occupational social class, area of residence, total physical activity, smoking, body mass index, history of cardiovascular disease, general practitioner diagnosis of hypertension, general practitioner diagnosis of type 2 diabetes, atrial fibrillation, heart failure, history of cancer, pulmonary disease and chronic kidney disease. Abbreviation: PIP, potentially inappropriate prescriptions; PTE, percent of exposure effect (macro SAS: https://www.hsph.harvard.edu/donna-spiegelman/software/mediate).

Trends in polypharmacy between 2005 and 2020 among older Moli-sani participants using data from the regional drug register are shown in Supplementary Table S7. Considering the variation in this exposure during the follow-up period, we repeated survival analyses, which showed that the hazard increased for all outcomes studied (Supplementary Table S8).

Stratified analyses by sex, age groups (65–75 years, ≥75 years) and education (low, high) showed no difference in the association between polypharmacy and the primary outcomes (Supplementary Table S9). On the other hand, stratification for a history of CVD showed that the higher hazard was mostly evident in individuals without a previous event of CVD (P-value for interaction 0.011 for all-cause mortality). Supplementary Table S10 reports the same analyses with CVD mortality and hospitalizations as outcomes, showing that older adults aged 65–75 years at baseline had a higher hazard than those ≥75 years (P-value for interaction = 0.039 and 0.022, respectively). It should be noted that individuals without multimorbidity at baseline showed the same hazards for all outcomes compared to those with two or more comorbidities (Supplementary Tables S9, S10).

## Discussion

In a general elderly population in Southern Italy, we observed that polypharmacy therapy was associated with a higher hazard of all-cause and specific (mainly cardiovascular) mortality and hospitalization. The results remained consistent after adjusting for a number of variables, including lifestyle habits, socioeconomic status, and various comorbidities, and after employing several statistical approaches and sensitivity analyses to minimize potential confounding factors and biases. However, we remain aware of the inherent difficulty in establishing a clear causal relationship between the exposure of interest and the outcome in observational studies.

Additionally, when the association between the polypharmacy regimen and the total number of hospitalizations and the total hospital days accrued during follow-up was investigated, an increase in the all-cause hospitalization “burden” on the National Health Service was observed in those on polypharmacy. When the variation in therapy regimen during follow-up was considered, the hazard of polytherapy increased for all outcomes.

Numerous studies have indicated that the use of multiple medications is associated with a wide range of adverse clinical events [13–15, 29–31]. However, it is difficult to understand whether the increased risk is due to the poor health status that required the prescription of medications or to the prescription of multiple medications *per se*. Moreover, despite rigorous adjustment for comorbidities, confounding by disease may persist in observational studies. We used propensity score analysis as an additional approach to control for residual confounding by comorbidities, which showed similar results. Moreover, stratification for CVD history showed that the higher hazard was mostly evident in individuals without a previous CVD event, strongly suggesting that polypharmacy may represent a health hazard beyond the confounding effect of comorbidities.

Interestingly, older adults without multimorbidity showed similar harms from polypharmacy compared to those with multimorbidity. This highlights that the risks associated with the use of multiple medications are not limited to those with multiple health conditions.

Our results are in line with previous studies [29–32]. The English Longitudinal Study of Ageing (6,295 individuals, aged ≥50 years), observed that, over a 6-year follow-up period, both polypharmacy (5–9 medications) and heightened polypharmacy (10+) were associated with a higher risk of all-cause mortality and CVD mortality, whereas cancer mortality was only related to heightened polypharmacy [30]. Chang TI et al., analyzing a large cohort of Korean older community-indwelling individuals (more than 3 million individuals, aged ≥65 years), found a graded association between the number of medications and the risk of adverse clinical outcomes and in particular, polypharmacy was associated with a significantly higher risk of hospitalization and mortality [29].

Several mechanisms may explain the relationship between polypharmacy and poor health status or increased mortality. Older individuals are more susceptible to serious adverse drug events due to age-related physiological changes that heighten the body’s sensitivity to drug effects. The harmful effect of polypharmacy on health and survival could be explained by the cumulative effects of multiple medications on the renal or hepatic system, which trigger a cascade of interactions in elderly individuals already suffering from multiple comorbidities [14]. Systematic reviews have reported that reducing specific classes of medications may reduce adverse events and improve quality of life [33–35].

Finally, polypharmacy is potentially harmful because it increases the possibility of inappropriate prescriptions. Therefore, we specifically evaluated whether PIP could potentially mediate the association between polypharmacy and poor health. Our longitudinal analyses showed that PIP, which are quite common among older adults receiving multiple drug prescriptions, are important mediators of the association between polypharmacy and key health outcomes. These results are supported by the finding that among individuals on polypharmacy, those without severe multimorbidity had the same polypharmacy burden as those with multimorbidity. As the number of individuals taking multiple medications on a regular basis increases, strategies to address the challenges related to polypharmacy burden are needed [35, 36].

The results of our study do not call into question the efficacy of any individual medication (among those examined in the study). This is because our study design, particularly the reference group used, was not intended to assess the efficacy of a single medication. Such efficacy evaluations should be conducted for each medication in its appropriate setting, such as hypertensive patients with or without the specific antihypertensive drug being tested, etc. However, this scenario does not align with the scope of our study. Clinical guidelines typically rely on evidence derived from randomized clinical trials or meta-analyses. However, these sources frequently exhibit bias due to the exclusion or inadequate representation of older individuals, particularly those with multiple health conditions and undergoing multiple medication treatments [37, 38].

Improving drug precription for older adults is a global priority for all healthcare systems. The majority of older individuals are cared for by general practitioners who may have inadequate expertise in geriatrics and multimorbidity particularly in polypharmacy management. Our findings strongly support the need for public action to improve the culture of appropriate prescribing and specific research and clinical pharmacological knowledge for the elderly population.

### Strengths and Limitations

A strength of this study is that it is derived from a large population-based adult cohort with a large panel of potential confounders available to adjust for some factors that may be involved in the hypothesized causal pathway between polypharmacy and the studied health outcomes; additionally, a propensity score approach was used to control for residual confounding by comorbidities.

Our study shares a major limitation of several previous epidemiologic studies in that we had only a single baseline measure of covariates included in the multivariable model or propensity score. However, we collected polypharmacy conditions during follow-up, which allowed us to perform survival analyses with a time-varying exposure. Unfortunately, it was not possible to provide a detailed breakdown of the potentially inappropriate prescriptions according to specific criteria. The current results reflect the overall frequency of PIP based on the AGS Beers Criteria^®^.

The data were collected in a Mediterranean region between Central and Southern Italy, so caution is needed in generalizing the results. However, the main characteristics of the Moli-sani sample are comparable to those of the Italian Cardiovascular Epidemiological Observatory, making it representative of the Italian population [39].

In conclusion, older adults on polypharmacy had a higher hazard of mortality and hospitalization for all causes and specifically for CVD. The study shows that in a general older population PIP are important mediators of the association between polypharmacy therapy and poor health outcomes. Nevertheless, our findings show that among individuals on polypharmacy therapy, those without multimorbidity appear to suffer the same “multiple medication-related harms” as those with multimorbidity.

The main concern in addressing the challenge of “medication without harm” should be the assessment of the appropriateness of drug prescribing and monitoring for adverse effects potentially linked to multiple medications.

## Data Availability

The data underlying this article will be made available upon reasonable request to the corresponding author. The data are stored in an institutional repository (https://repository.neuromed.it) and access is restricted by the ethical approvals and the legislation of the European Union.
