# Supplementary material for: Polypharmacy in Older Adults: The Hazard of Hospitalization and Mortality is Mediated by Potentially Inappropriate Prescriptions, Findings From the Moli-sani Study
Source: Int J Public Health. 2024 Oct 24;69:1607682. doi: 10.3389/ijph.2024.1607682 (PMC11540657; doi:10.3389/ijph.2024.1607682)
Supplement: Supplementary file 1 [file DataSheet1.doc]

**SUPPLEMENTAL MATERIAL**

Polypharmacy in Older Adults: The Hazard of Hospitalization and Mortality Is Mediated by Potentially Inappropriate Prescriptions, Findings from the Moli-sani Study

**Table S1.** Code for cause-specific admissions to hospital.

| **Cardiovascular Disease** | If **MDC** was coded as 05  *or*  the primary diagnosis of admission to hospital was coded as **ICD-9:** 390-459 and **MDC** was coded: 01 or 05. |
| --- | --- |
| **Ischemic heart disease** | If the primary diagnosis of admission to hospital was coded as **ICD-9:** 410-414  *or*  surgical procedure was coded as **ICD-9:** 36.0-36.1. |
| **Cerebrovascular Disease** | If the primary diagnosis of admission to hospital was coded as **ICD-9:** 430-434,436-438  *or*  surgical procedure was coded as **ICD-9:** 38.12 |

All HDRs were coded using the Italian Diagnosis Related Groups classification (version 24^th^), that includes Major Diagnostic Categories. Moreover, in the HDRs, the primary and secondary diagnoses for admission to hospital were reported using the ICD-9 codes.

**Abbreviations:** **ICD-9**: 9^th^ version of the International Classification of Diseases; **MDC**: Major Diagnostic Category *(MDC:* ***01*** *- Diseases & Disorders of the Nervous System;* ***05*** *- Diseases & Disorders of the Circulatory System.*

**Appendix S1.** Risk factor and comorbidity assessment.

At baseline visit, structured questionnaires to collect personal and clinical information, including socioeconomic status, physical activity, physio-pathological medical history, risk factors for CVD and/or tumour, and drug use, and dietary habits were administered.

History of CVD (including angina, myocardial infarction, revascularization procedures, cerebrovascular events and peripheral artery disease) and cancer were self-reported by participants during the baseline visit. All medical history reported were confirmed if participant: 1) reported the date of admission to the hospital; b) reported drug use for the specific disease; c) presented medical records of disease diagnosis. Information on physician-made diagnoses of hypertension, hypercholesterolemia, heart failure, atrial fibrillation, liver disorder, kidney disease, lung disease, gastrointestinal disease, neurodegenerative diseases (Parkinson’s disease, Alzheimer’s disease), depression, osteoporosis and pathologies of the thyroid gland were also collected.

Urban or rural environments were defined on the basis of the urbanization level as described by the European Institute of Statistics (EUROSTAT definition) and obtained by the tool ‘Atlante Statistico dei Comuni’ provided by the Italian National Institute of Statistics [1].

Educational attainment was based on the highest qualification attained and categorized as low (up to lower secondary school; approximately ≤ 8 years of study) or high (upper secondary education or higher; approximately ≥ 9 years of study). Household income was a three-level variable (< 40,000; ≥ 40,000 Euros/year), with missing values collapsed into a non-respondent category.

Physical activity was assessed by a structured questionnaire (24 questions on working time, leisure time, weekly walking and sport participation) and expressed as daily energy expenditure in metabolic equivalent task-hours (MET-hour) [2].

Subjects were classified as “non-smokers” if they had smoked less than 100 cigarettes in their lifetime, or they had never smoked cigarettes, as “current smokers” those who reported having smoked at least 100 cigarettes in their lifetime and still smoked or had quit smoking within the preceding year, and “former smokers” if they had smoked cigarettes in the past and had stopped smoking for at least one year. Body mass index (BMI), calculated as kg/m^2^ and obesity was defined ad BMI ≥ 30 kg/m^2^.

Food intake was assessed by the validated Italian EPIC food frequency questionnaire [3]. Adherence to the Mediterranean diet was assessed with the Mediterranean Diet Score (MDS), which was obtained by assigning 1 point to healthy foods (fruits and nuts, vegetables, legumes, fish, cereals, monounsaturated to saturated fats ratio) whose consumption was above the sex-specific medians of intake of the population; foods presumed to be detrimental (meat and dairy products) were scored positively if the consumption was below the median. All other intakes received 0 points. For ethanol, men who consumed 10–50 g/d and women who consumed 5–25 g/d received 1 point; otherwise, the score was 0. The MDS ranged from 0 to 9 (the latter reflecting maximal adherence) and was used either as continuous variable [3-4].

The average volume of alcohol consumed during the year before enrolment was assessed by the validated Italian EPIC food frequency questionnaire, complemented by specific supplementary questions. In calculating the amount of alcohol consumed, we assumed that an alcoholic unit is equivalent to 120 ml wine, 330 ml beer or 40 ml liquor, and contains 12 g ethanol. At baseline, participants who reported current abstention but had ever consumed alcohol were classified as former drinkers. Occasional drinkers included subjects who reported consumption less than 2.5 alcoholic units/month [5].

**REFERENCE**

1. ISTAT: Istituto Nazionale di Statistica, Atlante statistico dei comuni. Edizione 2014. Available at: https://www.istat.it/it/archivio/113712 (Accessed September 2024).
2. Ainsworth BE, Haskell WL, Whitt MC. Compendium of physical activities: an update of activity codes and MET intensities. Med Sci Sports Exerc 2000; 32(Suppl): S498–S504.
3. Bonaccio M, Di Castelnuovo A, Costanzo S, et al. Association of a traditional Mediterranean diet and non-Mediterranean dietary scores with all-cause and cause-specific mortality: prospective findings from the Moli-sani Study. Eur J Nutr. 2021 Mar;60(2):729-746.
4. Trichopoulou A, Costacou T, Bamia C, Trichopoulos D. Adherence to a Mediterranean diet and survival in a Greek population. N Engl J Med 2003;348:2599–608.
5. Costanzo S, Mukamal KJ, Di Castelnuovo A, et al. Alcohol consumption and hospitalization burden in an adult Italian population: prospective results from the Moli-sani study. Addiction. 2019 Apr;114(4):636-650.

**Figure S1.A** The unadjusted standardized differences and standardized differences adjusted by propensity scores between No Chronic Polypharmacy Therapy and No Polypharmacy Therapy individuals for each variable included in the propensity score.

**
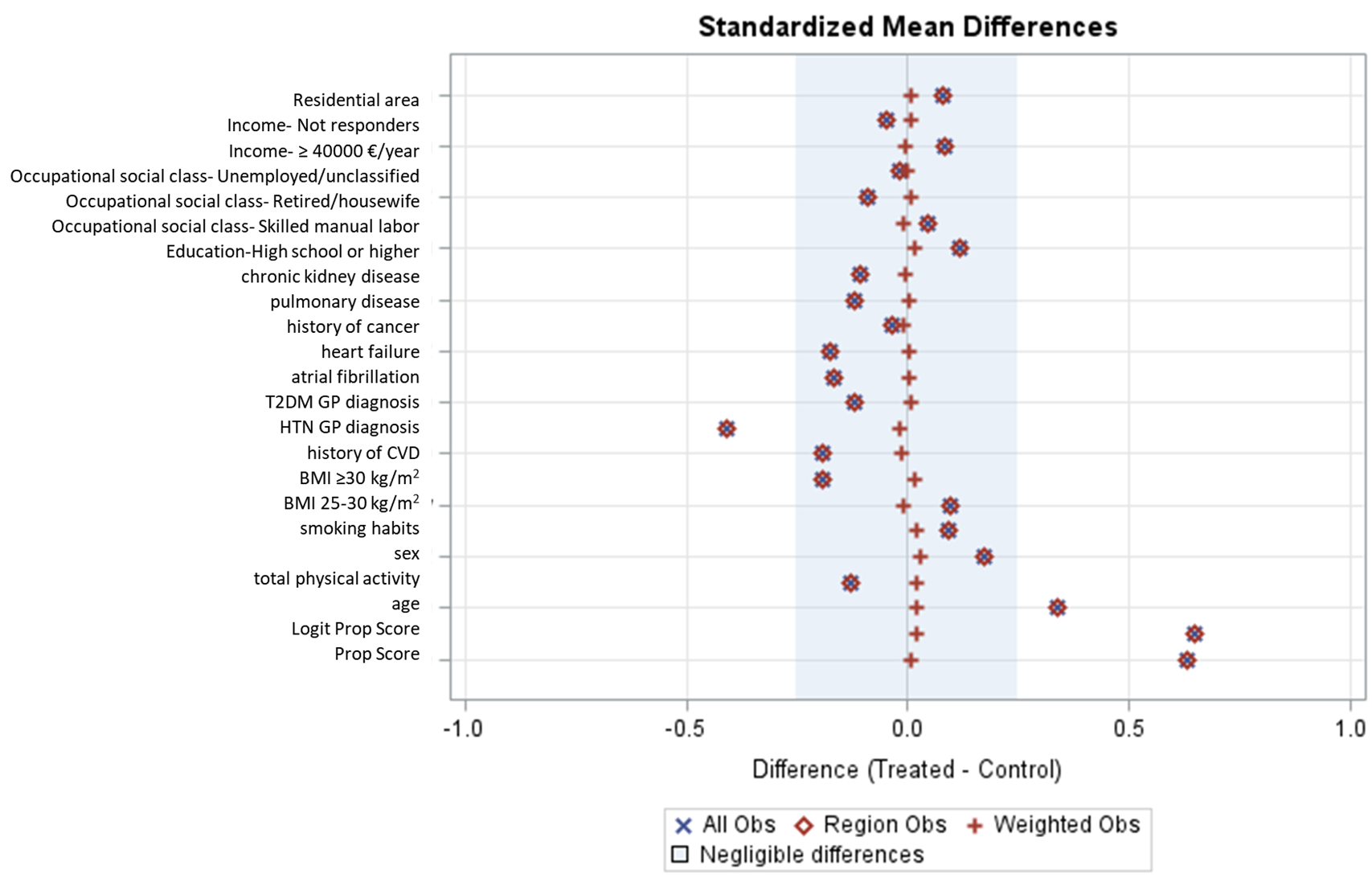
**

**Figure S1.B** The unadjusted standardized differences and standardized differences adjusted by propensity scores between Chronic Polypharmacy Therapy and No Polypharmacy Therapy individuals for each variable included in the propensity score.

**
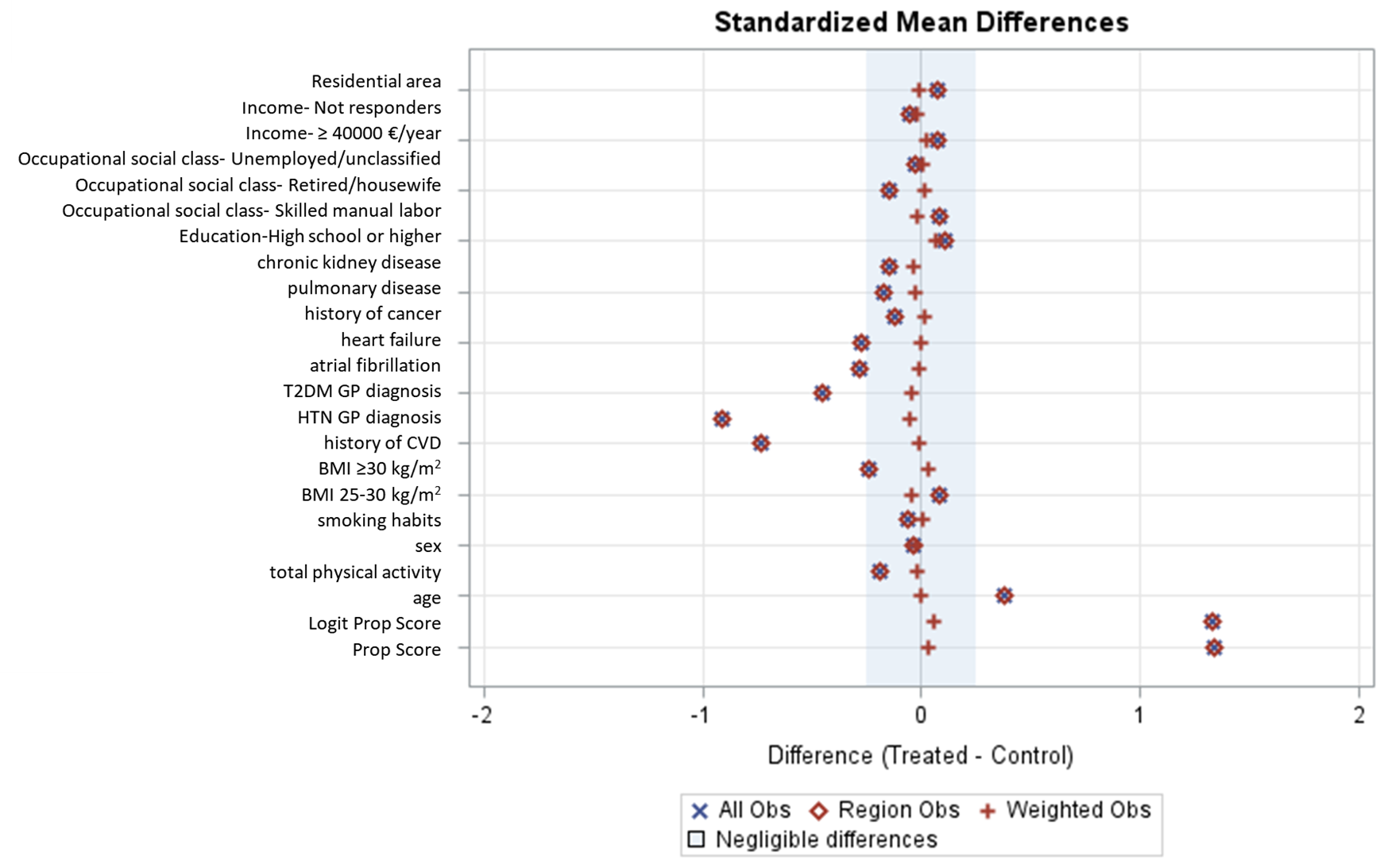
**

**Table S2.** Prevalence of the demographic and lifestyle characteristics according to polypharmacy therapy in the elderly of the Moli-sani Study (N= 5,631), (Italy, 2005-2010).

|  | **No Polypharmacy Therapy** | **No Chronic Polypharmacy Therapy** | **Chronic Polypharmacy Therapy** | **P**  **value*** |
| --- | --- | --- | --- | --- |
| **N (%)** | 2,934 (52.1) | 1,063 (18.9) | 1,634 (29.0) |  |
| **Age, years** | 71.5 (5.1) | 73.3 (5.5) | 73.5 (5.6) | <.0001 |
| **Age ≥ 75 years, %** | 23.0 | 34.0 | 36.1 | <.0001 |
| **Men, %** | 52.0 | 43.4 | 53.7 | <.0001 |
| **Residential area** |  |  |  | 0.073 |
| *Rural* | 32.4 | 36.2 | 35.9 |  |
| *Urban* | 67.6 | 63.8 | 64.1 |  |
| **Marital status, %** |  |  |  | 0.29 |
| *Married/cohabiting* | 76.7 | 73.1 | 74.1 |  |
| *Single/separated /divorced* | 4.5 | 4.4 | 3.4 |  |
| *Widower* | 18.8 | 22.5 | 22.5 |  |
| **Education, %** |  |  |  | 0.0001 |
| *Up to lower secondary school* | 74.0 | 79.1 | 78.6 |  |
| *High school or higher* | 26.0 | 20.9 | 21.4 |  |
| **Occupational social class** |  |  |  | 0.0013 |
| *Skilled, nonmanual labor* | 4.0 | 2.4 | 1.8 |  |
| *Skilled manual labor* | 3.0 | 2.3 | 1.7 |  |
| *Retired/housewife* | 92.7 | 94.9 | 96.1 |  |
| *Unemployed/unclassified* | 0.3 | 0.4 | 0.4 |  |
| **Income, %** |  |  |  | 0.53 |
| *< 40000 €/year* | 53.1 | 52.6 | 52.4 |  |
| *≥ 40000 €/year* | 6.6 | 4.7 | 4.8 |  |
| *Not responders* | 40.3 | 42.7 | 42.8 |  |
| **Childhood SES** |  |  |  | 0.0019 |
| *Low* | 22.2 | 22.0 | 25.3 |  |
| *Low-medium* | 51.3 | 54.4 | 53.1 |  |
| *Medium* | 23.8 | 20.9 | 19.3 |  |
| *High* | 2.7 | 2.8 | 2.4 |  |
| **Smoking habits, %** |  |  |  | 0.0002 |
| *Never smoker* | 55.1 | 59.6 | 52.0 |  |
| *Smoker* | 12.1 | 9.4 | 8.0 |  |
| *Ex-smoker* | 32.8 | 31.0 | 40.0 |  |
| **Total Physical Activity***, Met-h/day* | 41.6 (5.0) | 41.0 (4.0) | 40.8 (3.1) | <.0001 |
| **Mediterranean diet score** | 4.6 (1.6) | 4.4 (1.6) | 4.3 (1.6) | 0.13 |
| **Caloric intake***, kcal/day* | 1927.3 (590) | 1842 (579) | 1777 (529) | <.0001 |
| **Alcohol drinking patterns** |  |  |  | 0.029 |
| *Lifetime abstainer* | 20.0 | 26.4 | 24.9 |  |
| *Former drinker* | 5.6 | 6.2 | 6.4 |  |
| *Occasional drinker* | 4.4 | 4.7 | 5.6 |  |
| *Regular drinker* | 70.0 | 62.7 | 63.1 |  |
| *<12 gr/day* | 16.3 | 15.5 | 15.5 |  |
| *12-24 gr/day* | 18.4 | 17.0 | 18.8 |  |
| *≥24 gr/day* | 35.3 | 30.2 | 28.8 |  |

Values are reported as means with standard deviation (SD) for continuous variables and percentage for categorical variables. *P value adjusted for age and sex.

**Table S3.** Prevalence of chronic degenerative diseases according to polypharmacy therapy in the elderly population of the Moli-sani Study (N= 5,631), (Italy, 2005-2010).

|  | **No Polypharmacy Therapy** | **No Chronic Polypharmacy Therapy** | **Chronic Polypharmacy Therapy** | **P value*** |
| --- | --- | --- | --- | --- |
| **N (%)** | 2,934 (52.1) | 1,063 (18.9) | 1,634 (29.0) |  |
| **# chronic diseases** |  |  |  | <.0001 |
| *0* | 23.0 | 9.2 | 1.4 |  |
| *1* | 37.3 | 29.4 | 14.0 |  |
| *2* | 25.2 | 31.0 | 29.1 |  |
| *3* | 10.9 | 20.5 | 29.1 |  |
| *≥4* | 3.6 | 9.8 | 26.4 |  |
| ***Risk factors for major chronic degenerative diseases*** | |  |  |  |
| **BMI, kg/m^2^** | 28.3 (4.4) | 29.3 (4.8) | 29.8 (4.9) | <.0001 |
| **Obese, BMI ≥30 kg/m^2^** | 31.3 | 40.7 | 42.7 | <.0001 |
| **Hypertension, GP diagnosis** | 45.0 | 65.0 | 84.8 | <.0001 |
| **Diabetes, GP diagnosis** | 7.7 | 11.2 | 24.0 | <.0001 |
| **Hypercholesterolemia, GP diagnosis** | 26.7 | 34.0 | 47.4 | <.0001 |
| **Metabolic Syndrome** | 32.0 | 37.8 | 48.9 | <.0001 |
| ***Age related diseases*** |  |  |  |  |
| **History of CVD** | 6.1 | 11.7 | 34.2 | <.0001 |
| **Heart failure** | 0.5 | 2.6 | 4.8 | <.0001 |
| **Atrial fibrillation** | 4.6 | 8.8 | 12.4 | <.0001 |
| **History of Cancer** | 5.7 | 6.6 | 8.9 | 0.0029 |
| **Liver disorders** | 4.7 | 4.4 | 5.3 | 0.43 |
| **Gastrointestinal disorders** | 11.8 | 14.5 | 17.0 | <.0001 |
| **Lung disease** | 4.1 | 6.9 | 8.4 | <.0001 |
| **Chronic kidney disease** | 0.1 | 0.7 | 1.2 | <.0001 |
| **Alzheimer** | 0.1 | 0.3 | 0.4 | 0.30 |
| **Parkinson Disease** | 0.3 | 0.9 | 0.7 | 0.087 |
| **Depression** | 8.7 | 12.5 | 15.1 | <.0001 |
| **Osteoporosis** | 12.3 | 19.1 | 17.2 | <.0001 |
| **Pathologies of the thyroid gland** | 12.7 | 18.9 | 23.4 | <.0001 |

Values are reported as means with standard deviation (SD) for continuous variables and percentage for categorical variables. *P value adjusted for age and sex. Abbreviation: BMI body mass index; CVD cardiovascular disease.

**Table S4.** Distribution of the total number of hospitalizations and total number of hospital days accrued during follow-up for all-cause, cardiovascular, ischemic heart and, cerebrovascular disease hospitalizations in the elderly of the Moli-sani Study (N = 5,631), (Italy, 2005-2010).

|  | **Hospitalizations** | **Hospital days** |
| --- | --- | --- |
| **All-cause hospitalization** |  |  |
| At least one during follow-up | 4,342 | - |
| Sum | 15,161 | 131,820 |
| Median (IQR) in whole sample | 2 (1-4) | 13 (2-33) |
| Median (IQR) in hospitalized individuals | 3 (1-4) | 20 (9-41) |
| **Cardiovascular hospitalization** |  |  |
| At least one during follow-up | 2,463 | - |
| Sum of events | 5,559 | 44,512 |
| Median (IQR) in whole sample | 0 (0-1) | 0 (0-10) |
| Median (IQR) in hospitalized individuals | 2 (1-23) | 12 (6-22) |
| **Ischemic heart disease hospitalization** |  |  |
| At least one during follow-up | 615 | - |
| Sum of events | 1,017 | 7,461 |
| Median (IQR) in whole sample | 0 (0-0) | 0 (0-0) |
| Median (IQR) in hospitalized individuals | 1 (1-2) | 8 (4-16) |
| **Cerebrovascular hospitalization** |  |  |
| At least one during follow-up | 705 | - |
| Sum of events | 875 | 8,311 |
| Median (IQR) in whole sample | 0 (0-0) | 0 (0-0) |
| Median (IQR) in hospitalized individuals | 1 (1-1) | 8 (5-14) |

IQR: Interquartile range

**Table S5.** Incident rate ratio (95% Confidence Interval) for total number of hospitalizations and total number of hospital days accrued during follow-up for all-cause, cardiovascular, ischemic heart and, cerebrovascular disease hospitalizations, according to polypharmacy, in the elderly of the Moli-sani Study (N = 5,631), (Italy, 2005-2010).

|  | | **No Polypharmacy Therapy** | **No Chronic Polypharmacy Therapy** | **P value**  **NC-PT vs**  **No PT** | **Chronic Polypharmacy Therapy** | **P value**  **C-PT vs**  **No PT** | **P value**  **NC-PT vs**  **C-PT** |
| --- | --- | --- | --- | --- | --- | --- | --- |
| **N (%)** | | 2,934 (52.1) | 1,063 (18.9) |  | 1,634 (29.0) |  |  |
|  | **Total hospitalizations during follow-up** | | | | | | |
| **All-cause hospitalization** | |  |  |  |  |  |  |
| **Mean (SE)*** | | 2.1 (0.05) | 2.7 (0.09) |  | 3.7 (0.07) |  |  |
| **IRR (95% CI) model 1** | | Ref. | 1.29 (1.23-1.35) | <.0001 | 1.73 (1.67-1.80) | <.0001 | <.0001 |
| **IRR (95% CI) model 2** | | Ref. | 1.22 (1.17-1.28) | <.0001 | 1.50 (1.44-1.57) | <.0001 | <.0001 |
| **Cardiovascular hospitalization** | |  |  |  |  |  |  |
| **Mean (SE)*** | | 0.6 (0.03) | 1.0 (0.05) |  | 1.6 (0.04) |  |  |
| **IRR (95% CI) model 1** | | Ref. | 1.49 (1.38-1.61) | <.0001 | 2.47 (2.33-2.63) | <.0001 | <.0001 |
| **IRR (95% CI) model 2** | | Ref. | 1.33 (1.23-1.44) | <.0001 | 1.82 (1.70-1.95) | <.0001 | <.0001 |
| **Ischemic heart disease hospitalization** | | |  |  |  |  |  |
| **Mean (SE)*** | | 0.1 (0.01) | 0.2 (0.02) |  | 0.3 (0.02) |  |  |
| **IRR (95% CI) model 1** | | -1- | 1.55 (1.29-1.87) | <.0001 | 2.70 (2.35-3.10) | <.0001 | <.0001 |
| **IRR (95% CI) model 2** | | -1- | 1.40 (1.16-1.69) | 0.0005 | 1.75 (1.48-2.06) | <.0001 | 0.019 |
| **Cerebrovascular hospitalization** | |  |  |  |  |  |  |
| **Mean (SE)*** | | 0.1 (0.01) | 0.2 (0.01) |  | 0.2 (0.01) |  |  |
| **IRR (95%CI) model 1** | | Ref. | 1.29 (1.07-1.55) | 0.0066 | 1.59 (1.37-1.83) | <.0001 | 0.023 |
| **IRR (95%CI) model 3** | | Ref. | 1.26 (1.04-1.51) | 0.017 | 1.46 (1.23-1.74) | <.0001 | 0.12 |
|  | **Total hospital days during follow-up** | | | | | | |
| **All-cause hospitalization** | |  |  |  |  |  |  |
| **Mean (SE) for each hospitalization*** | | 18.7 (0.54) | 23.6 (0.89) |  | 31.7 (0.72) |  |  |
| **IRR (95% CI) model 1b** | | Ref. | 1.18 (1.16-1.19) | <.0001 | 1.22 (1.21-1.24) | <.0001 | <.0001 |
| **IRR (95% CI) model 2b** | | Ref. | 1.14 (1.12-1.15) | <.0001 | 1.15 (1.13-1.16) | <.0001 | 0.14 |
| **Cardiovascular hospitalization** | |  |  |  |  |  |  |
| **Mean (SE) for each hospitalization*** | | 5.2 (0.29) | 7.5 (0.48) |  | 13.0 (0.39) |  |  |
| **IRR (95% CI) model 1b** | | Ref. | 1.26 (1.22-1.29) | <.0001 | 1.33 (1.30-1.36) | <.0001 | <.0001 |
| **IRR (95% CI) model 2b** | | Ref. | 1.20 (1.17-1.24) | <.0001 | 1.21 (1.17-1.24) | <.0001 | 0.86 |
| **Ischemic heart disease hospitalization** | | |  |  |  |  |  |
| **Mean (SE) for each hospitalization*** | | 0.8 (0.10) | 1.4 (0.17) |  | 2.2 (0.14) |  |  |
| **IRR (95% CI) model 1b** | | Ref. | 1.67 (1.56-1.79) | <.0001 | 1.84 (1.75-1.94) | <.0001 | 0.0031 |
| **IRR (95% CI) model 2b** | | Ref. | 1.45 (1.35-1.55) | <.0001 | 1.24 (1.17-1.32) | <.0001 | <.0001 |
| **Cerebrovascular hospitalization** | |  |  |  |  |  |  |
| **Mean (SE) for each hospitalization*** | | 1.3 (0.11) | 1.7 (0.18) |  | 1.8 (0.15) |  |  |
| **IRR (95% CI) model 1b** | | Ref. | 1.10 (1.04-1.17) | 0.0008 | 0.77 (0.73-0.82) | <.0001 | <.0001 |
| **IRR (95% CI) model 2b** | | Ref. | 0.92 (0.86-0.97) | 0.0054 | 0.60 (0.56-0.64) | <.0001 | <.0001 |

Incident rate ratios of multiple re-admissions to hospital for according to polypharmacy categories were calculated using the Poisson regression analysis. Additionally, the association between polypharmacy categories and total number of hospital days during follow-up was tested using a multivariable Poisson regression model, with adjustment for the observed length of follow-up.

*Mean and Standard Error adjusted for age, sex and time (person-time censored only by death or the end of follow-up).

Model 1: adjusted for age, sex and time; Model 2: as Model 1 further adjusted for educational levels, income, occupational social class, residential area, total physic activity, smoking, body mass index, history of cardiovascular disease, general practitioner diagnosis of hypertension, general practitioner diagnosis of type 2 diabetes, atrial fibrillation, heart failure, history of cancer, pulmonary disease and chronic kidney disease; Model Xb: as model X plus number of hospitalizations during follow-up.

Abbreviation: C-PT chronic polypharmacy therapy; NC-PT non-chronic polypharmacy therapy; No PT no polypharmacy therapy; CI confidence interval; IRR Incident rate ratios; SE standard error.

**Table S6.** Role of potentially inappropriate prescriptions in the relationship between polypharmacy therapy and main outcomes, (Italy, 2005-2010).

|  | **No PT,**  **No PIP** | **No PT,**  **and PIP** | **NC-PT**  **No PIP** | **NC-PT**  **and PIP** | **C-PT,**  **No PIP** | **C-PT**  **and PIP** | **P value** |
| --- | --- | --- | --- | --- | --- | --- | --- |
| **N (%)** | 2,609 (46.3) | 325 (5.8) | 754 (13.4) | 309 (5.5) | 927 (16.5) | 707 (12.5) |  |
| **All-cause mortality** | | | | | | | |
| **Person Years** | 31,488 | 3,748 | 8,557 | 3,339 | 10,130 | 7,454 |  |
| **N of events (rate %)** | 709 (27.2) | 110 (33.9) | 279 (37.0) | 129 (41.8) | 419 (45.2) | 355 (50.2) |  |
| ***HR***  ***(95% CI)*** | Ref. | 1.26  (1.03-1.55) | 1.22  (1.05-1.40) | 1.34  (1.11-1.62) | 1.30  (1.14-1.49) | 1.40  (1.21-1.62) | <.0001 |
| **All-cause hospitalization** | | | | | | | |
| **Person Years** | 19,148 | 2,160 | 4,297 | 1,642 | 4,403 | 2,814 |  |
| **N of events (rate %)** | 1821 (69.8) | 238 (73.2) | 607 (80.5) | 254 (82.2) | 801 (86.4) | 621 (87.8) |  |
| ***HR***  ***(95% CI)*** | Ref. | 1.13  (0.98-1.29) | 1.39  (1.27-1.53) | 1.45  (1.27-1.66) | 1.55  (1.41-1.70) | 1.79  (1.62-1.98) | <.0001 |

Model adjusted for age, sex, educational levels, income, occupational social class, residential area, total physical activity, smoking, body mass index, history of cardiovascular disease, general practitioner diagnosis of hypertension, general practitioner diagnosis of type 2 diabetes, atrial fibrillation, heart failure, history of cancer, pulmonary disease and chronic kidney disease.

Abbreviation: C-PT chronic polypharmacy therapy; NC-PT non-chronic polypharmacy therapy; No PT no polypharmacy therapy; CI confidence interval; HR hazard ratio; PIP potentially inappropriate prescriptions.

**Table S7.** Trends in polypharmacy among the elderly Moli-sani participants using data from the regional register of drugs between 2005 and 2015, (Italy, 2005-2010).

| **Years** | **N**  **Moli-sani** | **C-PT or NC-PT, %** | | | |
| --- | --- | --- | --- | --- | --- |
|  |  | **Whole**  **sample** | **Age Classes, years** | | |
|  |  |  | **65-74** | **75-84** | **≥85** |
| **2005-2010*** | 5,631 | 47.9 | 43.6 | 57.5 | 69.2 |
| **2011** | 5,336 | 57.6 | 52.0 | 62.8 | 69.9 |
| **2012** | 5,214 | 59.2 | 52.6 | 63.4 | 71.6 |
| **2013** | 5,099 | 61.0 | 52.8 | 65.7 | 69.2 |
| **2014** | 4,956 | 62.2 | 53.9 | 65.4 | 68.8 |
| **2015** | 4,797 | 63.2 | 54.0 | 65.3 | 67.8 |
| **2016** | 4,603 | 65.2 | 55.1 | 65.8 | 71.0 |
| **2017** | 4,438 | 66.9 | 54.8 | 66.5 | 73.1 |
| **2018** | 4,272 | 67.5 | 57.2 | 65.8 | 74.0 |
| **2019** | 4,066 | 69.3 | 65.6 | 67.3 | 74.6 |
| **2020** | 3,830 | 68.7 | 64.6 | 67.3 | 72.7 |

*recruitment phase. The N of participants shown in this table considers those lost to follow-up and those who died during the years of observation.

**Table S8.** Hazard Ratio (95% Confidence Interval) for main outcomes according to Polypharmacy therapy as a time-varying variable (N = 5,631), (Italy, 2005-2010).

|  | **No PT** | **C-PT** | **P value** | **NC-PT**  **or C-PT** | **P value** |
| --- | --- | --- | --- | --- | --- |
| **N (%)** | 2,934 (52.1) | 1,634 (29.0) |  | 2,697 (47.9) |  |
| **All-cause mortality** |  |  |  |  |  |
| **HR (95%CI)** | Ref. | 1.34 (1.19-1.51) | <.0001 | 1.26 (1.14-1.39) | <.0001 |
| **HR (95%CI) + C-PT during FUP** | Ref. | 1.45 (1.28-1.66) | <.0001 | 1.43 (1.27-1.62) | <.0001 |
|  |  |  |  |  |  |
| **Cardiovascular mortality** |  |  |  |  |  |
| **HR (95%CI)** | Ref. | 1.57 (1.30-1.89) | <.0001 | 1.40 (1.19-1.65) | <.0001 |
| **HR (95%CI) + C-PT during FUP** | Ref. | 1.62 (1.30-2.02) | <.0001 | 1.56 (1.26-1.92) | <.0001 |
| **Cancer mortality** |  |  |  |  |  |
| **HR (95%CI)** | Ref. | 1.06 (0.84-1.34) | 0.61 | 1.13 (0.94-1.36) | 0.20 |
| **HR (95%CI) + C-PT during FUP** | Ref. | 1.29 (1.03-1.62) | 0.029 | 1.33 (1.08-1.64) | 0.0074 |
|  |  |  |  |  |  |
| **All-cause hospitalization** |  |  |  |  |  |
| **HR (95%CI)** | Ref. | 1.61 (1.49-1.75) | <.0001 | 1.50 (1.40-1.60) | <.0001 |
| **HR (95%CI) + C-PT during FUP** | Ref. | 1.60 (1.48-1.73) | <.0001 | 1.55 (1.44-1.66) | <.0001 |
| **Cardiovascular hospitalization** |  |  |  |  |  |
| **HR (95%CI)** | Ref. | 1.68 (1.51-1.85) | <.0001 | 1.50 (1.37-1.64) | <.0001 |
| **HR (95%CI) + C-PT during FUP** | Ref. | 1.77 (1.59-1.97) | <.0001 | 1.65 (1.50-1.82) | <.0001 |
| **Ischemic heart disease hospitalization** | |  |  |  |  |
| **HR (95%CI)** | Ref. | 1.64 (1.33-2.04) | <.0001 | 1.57 (1.30-1.89) | <.0001 |
| **HR (95%CI) + C-PT during FUP** | Ref. | 1.94 (1.54-2.45) | <.0001 | 1.91 (1.54-2.37) | <.0001 |
| **Cerebrovascular hospitalization** |  |  |  |  |  |
| **HR (95%CI)** | Ref. | 1.39 (1.14-1.70) | 0.0010 | 1.30 (1.10-1.54) | 0.0021 |
| **HR (95%CI) + C-PT during FUP** | Ref. | 1.45 (1.18-1.79) | 0.0004 | 1.41 (1.17-1.71) | 0.0004 |

Model adjusted for age, sex, educational levels, income, occupational social class, residential area, total physical activity, smoking, body mass index, history of cardiovascular disease, general practitioner diagnosis of hypertension, general practitioner diagnosis of type 2 diabetes, atrial fibrillation, heart failure, history of cancer, pulmonary disease and chronic kidney disease.

Abbreviation: C-PT chronic polypharmacy therapy; NC-PT non-chronic polypharmacy therapy; No PT no polypharmacy therapy; CI confidence interval; FUP follow-up;

**Table S9.** Hazard Ratio (95% Confidence Interval) for all-cause mortality and all-cause hospitalization according to polypharmacy therapy as a time-varying variable (n = 5,631), stratified by sex, age classes, education, history of CVD and presence of multimorbidity, (Italy, 2005-2010).

|  | **No PT** | **C-PT or NC-PT** | **P value** | **P for**  **interaction** |
| --- | --- | --- | --- | --- |
| **All-cause mortality** | | | |  |
| **Sex** |  |  |  | 0.29 |
| *Men* | -1- | 1.44 (1.23-1.68) | <.0001 |  |
| *Women* | -1- | 1.44 (1.18-1.76) | 0.0004 |  |
| **Age classes** |  |  |  | 0.42 |
| *65-75 years* | -1- | 1.45 (1.22-1.72) | <.0001 |  |
| *≥ 75 years* | -1- | 1.43 (1.20-1.72) | <.0001 |  |
| **Education** |  |  |  | 0.66 |
| *Low* | -1- | 1.41 (1.23-1.63) | <.0001 |  |
| *High* | -1- | 1.49 (1.15-1.92) | 0.0024 |  |
| **History of CVD** |  |  |  | 0.011 |
| *No* | -1- | 1.50 (1.32-1.71) | <.0001 |  |
| *Yes* | -1- | 0.88 (0.61-1.28) | 0.51 |  |
| **Multi-comorbidity** |  |  |  | 0.80 |
| *0-1* | -1- | 1.34 (1.14-1.57) | 0.0004 |  |
| *≥ 2* | -1- | 1.46 (1.20-1.78) | 0.0002 |  |
| **All-cause hospitalization** | | | |  |
| **Sex** |  |  |  | 0.013 |
| *Men* | -1- | 1.48 (1.35-1.63) | <.0001 |  |
| *Women* | -1- | 1.62 (1.46-1.79) | <.0001 |  |
| **Age classes** |  |  |  | 0.091 |
| *65-75 years* | -1- | 1.57 (1.44-1.70) | <.0001 |  |
| *≥ 75 years* | -1- | 1.50 (1.31-1.70) | <.0001 |  |
| **Education** |  |  |  | 0.91 |
| *Low* | -1- | 1.55 (1.44-1.68) | <.0001 |  |
| *High* | -1- | 1.52 (1.31-1.76) | <.0001 |  |
| **History of CVD** |  |  |  | 0.88 |
| *No* | -1- | 1.56 (1.45-1.68) | <.0001 |  |
| *Yes* | -1- | 1.58 (1.27-1.96) | <.0001 |  |
| **Multi-comorbidity** |  |  |  | 0.79 |
| *0-1* | -1- | 1.51 (1.38-1.65) | <.0001 |  |
| *≥ 2* | -1- | 1.54 (1.38-1.71) | <.0001 |  |

Model adjusted for age, sex, educational levels, income, occupational social class, residential area, total physical activity, smoking, body mass index, history of cardiovascular disease, general practitioner diagnosis of hypertension, general practitioner diagnosis of type 2 diabetes, atrial fibrillation, heart failure, history of cancer, pulmonary disease and chronic kidney disease.

Abbreviation: C-PT chronic polypharmacy therapy; NC-PT non-chronic polypharmacy therapy; No PT no polypharmacy therapy; CI confidence interval; CVD cardiovascular disease.

**Table S10.** Hazard ratio (95% Confidence Interval) for cardiovascular mortality and hospitalization, according to polypharmacy therapy as a time-varying variable (n = 5,631), stratified by sex, age classes, education, history of CVD and presence of multimorbidity, (Italy, 2005-2010).

|  | **No PT** | **C-PT or NC-PT** | **P value** | **P for**  **interaction** |
| --- | --- | --- | --- | --- |
| **Cardiovascular mortality** | | | |  |
| **Sex** |  |  |  | 0.81 |
| *Men* | -1- | 1.60 (1.20-2.13) | 0.0013 |  |
| *Women* | -1- | 1.45 (1.06-1.99) | 0.022 |  |
| **Age classes** |  |  |  | 0.039 |
| *65-75 years* | -1- | 1.80 (1.28-2.53) | 0.0007 |  |
| *≥ 75 years* | -1- | 1.38 (1.05-1.81) | 0.021 |  |
| **Education** |  |  |  | 0.41 |
| *Low* | -1- | 1.51 (1.19-1.92) | 0.0007 |  |
| *High* | -1- | 1.80 (1.12-2.88) | 0.015 |  |
| **History of CVD** |  |  |  | 0.26 |
| *No* | -1- | 1.45 (1.16-1.82) | 0.0014 |  |
| *Yes* | -1- | 2.66 (1.16-6.09) | 0.021 |  |
| **Multi-comorbidity** |  |  |  | 0.69 |
| *0-1* | -1- | 1.50 (1.12-1.99) | 0.0057 |  |
| *≥ 2* | -1- | 1.49 (1.07-2.06) | 0.017 |  |
| **Cardiovascular hospitalization** | | | |  |
| **Sex** |  |  |  | 0.088 |
| *Men* | -1- | 1.63 (1.42-1.85) | <.0001 |  |
| *Women* | -1- | 1.71 (1.47-1.98) | <.0001 |  |
| **Age classes** |  |  |  | 0.022 |
| *65-75 years* | -1- | 1.73 (1.54-1.95) | <.0001 |  |
| *≥ 75 years* | -1- | 1.50 (1.25-1.79) | <.0001 |  |
| **Education** |  |  |  | 0.94 |
| *Low* | -1- | 1.67 (1.49-1.87) | <.0001 |  |
| *High* | -1- | 1.65 (1.34-2.04) | <.0001 |  |
| **History of CVD** |  |  |  | 0.61 |
| *No* | -1- | 1.64 (1.48-1.83) | <.0001 |  |
| *Yes* | -1- | 1.84 (1.36-2.48) | <.0001 |  |
| **Multi-comorbidity** |  |  |  | 0.87 |
| *0-1* | -1- | 1.62 (1.42-1.84) | <.0001 |  |
| *≥ 2* | -1- | 1.66 (1.42-1.93) | <.0001 |  |

Model adjusted for age, sex, educational levels, income, occupational social class, residential area, total physical activity, smoking, body mass index, history of cardiovascular disease, general practitioner diagnosis of hypertension, general practitioner diagnosis of type 2 diabetes, atrial fibrillation, heart failure, history of cancer, pulmonary disease and chronic kidney disease.

Abbreviation: C-PT chronic polypharmacy therapy; NC-PT non-chronic polypharmacy therapy; No PT no polypharmacy therapy; CI confidence interval; CVD cardiovascular disease.

**Appendix S2 -** Moli-sani Study Investigators

The enrolment phase of the Moli-sani Study was conducted at the Research Laboratories of the Catholic University in Campobasso (Italy), the follow up of the Moli-sani cohort is being conducted at the Department of Epidemiology and Prevention of the IRCCS Neuromed, Pozzilli, Italy.

**Steering Committee:** Licia Iacoviello*^#^(Chairperson), Giovanni de Gaetano*, Maria Benedetta Donati*.

**Scientific Secretariat:** Chiara Cerletti* (Coordinator), Marialaura Bonaccio*, Americo Bonanni*, Simona Costanzo*, Amalia De Curtis*, Augusto Di Castelnuovo*, Alessandro Gialluisi*^#^, Francesco Gianfagna^§^°, Mariarosaria Persichillo*, Teresa Di Prospero* (Secretary).

**Safety and Ethical Committee:** Jos Vermylen (Catholic University, Leuven, Belgio) (Chairperson), Renzo Pegoraro (Pontificia Accademia per la Vita, Roma, Italy), Antonio Spagnolo (Catholic University, Roma, Italy).

**External Event Adjudicating Committee**: Deodato Assanelli (Brescia, Italy), Livia Rago (Campobasso, Italy).

**Baseline and Follow-up Data Management:** Simona Costanzo* (Coordinator), Marco Olivieri (Campobasso, Italy), Sabatino Orlandi*, Teresa Panzera*.

**Data Analysis:** Augusto Di Castelnuovo* (Coordinator), Marialaura Bonaccio*, Simona Costanzo*, Simona Esposito*, Alessandro Gialluisi*^#^, Anwal Ghulam*, Francesco Gianfagna^§^°, Antonietta Pepe*, Emilia Ruggiero*, Sukshma Sharma*.

**Biobank, Molecular and Genetic Laboratory:** Amalia De Curtis* (Coordinator), Concetta Civitillo*, Alisia Cretella*, Sara Magnacca*, Fabrizia Noro*.

**Recruitment Staff:** Mariarosaria Persichillo* (Coordinator), Francesca Bracone*, Fiorella De Rita (CuoreSano ETS, Campobasso), Giuseppe Di Costanzo*, Sabrina Franciosa*, Martina Morelli*, Teresa Panzera*.

**Communication and Press Office:** Americo Bonanni*.

**Regional Institutions:** Direzione Generale per la Salute - Regione Molise; Azienda Sanitaria Regionale del Molise (ASReM, Italy); Agenzia Regionale per la Protezione Ambientale del Molise (ARPA Molise, Italy); Molise Dati Spa (Campobasso, Italy); Offices of vital statistics of the Molise region.

**Hospitals:** Presidi Ospedalieri ASReM: Ospedale A. Cardarelli – Campobasso, Ospedale F. Veneziale – Isernia, Ospedale San Timoteo - Termoli (CB), Ospedale Ss. Rosario - Venafro (IS), Ospedale Vietri – Larino (CB), Ospedale San Francesco Caracciolo - Agnone (IS); Casa di Cura Villa Maria –Campobasso; Responsible Research Hospital - Campobasso; IRCCS Neuromed - Pozzilli (IS).

***Department of Epidemiology and Prevention, IRCCS Neuromed, Pozzilli, Italy

^#^Department of Medicine and Surgery, LUM University “Giuseppe Degennaro”, Casamassima, Italy

^§^Mediterranea Cardiocentro, Napoli, Italy

°Department of Medicine and Surgery, University of Insubria, Varese, Italy

*Moli-sani Study Past Investigators are available at* [*https://www.moli-sani.org/?page_id=173*](https://www.moli-sani.org/?page_id=173)
